# Supplementary material for: Composition and Function of Telomerase—A Polymerase Associated with the Origin of Eukaryotes
Source: Biomolecules. 2020 Oct 8;10(10):1425. doi: 10.3390/biom10101425 (PMC7658794; doi:10.3390/biom10101425)
Supplement: Supplementary file 1 [file biomolecules-10-01425-s001.pdf]

Table S1. Telomere repeats in representative species.

Telomere repeat	Representative species	Reference
**TSAR**		
TTAGGG	*Ectocarpus siliculosus* (Stramenopiles)	[1] and references herein
TTTAGGG	*Phytophthora infestans* (Oomycetes)	[1] and references herein
TTTTGGGG	*Euplotes aediculatus* (Ciliata)	[1] and references herein
TTGGGG	*Tetrahymena* (Ciliata)	[1] and references herein
TTTGGG	*Chilodonella uncinata* (Ciliata)	[1] and references herein
TTTTAGGG	*Theileria annulata* (Apicomplexa)	[1] and references herein
TTTAGG	*Cryptosporidium parvum Iowa II* (Apicomplexa)	[1] and references herein
TTAGG	*Aurantiochytrium limacinum* (Stramenopiles)	[1] and references herein
**Haptista**		
TTAGGG	*Emiliania huxleyi* (Haptophyta)	[1] and references herein
**Cryptista**		
TTTAGGG	*Guillardia theta* (Cryptophyceae)	[1] and references herein
**Archaeplastida**		
TTTAGGG	*Arabidopsis thaliana* (Brassicaceae)	[2]
TTAGGG	*Asparagus officinalis* (Asparagales)	[3]
TTTTAGGG	*Chlamydomonas reinhardtii* (Chlorophyceae)	[4]
TTTTAGG	*Klebsormidium subtilissimum* (Charophyta)	[1]
TTCAGG/TTTCAGG	*Genlisea hispidula* (Lentibulariaceae)	[5]
CTCGGTTATGGG	*Allium cepa* (Amaryllidaceae)	[6]
AATGGGGGG	*Cyanidioschyzon merolae* (Rhodophyta)	[7,8]
TTTTTTAGGG	*Cestrum elegans* (Solanaceae)	[9]

**Amorphea**		
TTAGGG	*Homo sapiens* (Animalia)	[10]
TTAGG	*Bombyx mori* (Insects)	[11]
TTAGGC	*Ascaris lumbricoides* (Nematodes)	[12]
TCAGG	*Tribolium castaneu*m (Anthropoda)	[13]
TTGCA	*Parascaris univalens* (Nematoda)	[1] and references herein
TGTGGG	*Bdelloidea* (Rotifera)	[1] and references herein
TAAGGG	*Polysphodylium pallidum* (Amoebozoa)	[1] and references herein
**Discoba**		
TTAGGG	*Andalucia godoyi* (Jakobida)	[1] and references herein
**Metamonada**		
TAGGG	*Giardia lamblia* (Fornicata)	[1] and references herein
**Malawimonadida**		
TTAGGG	*Malawimonas californiana* (Malawimonadida)	[1] and references herein

1. Fulnečková, J.; Ševčíková, T.; Fajkus, J.; Lukešová, A.; Lukeš, M.; Vlček, Č.; Lang, B.F.; Kim, E.; Eliáš, M.; Sýkorová, E. A Broad Phylogenetic Survey Unveils the Diversity and Evolution of Telomeres in Eukaryotes. *Genome Biology and Evolution* **2013**, *5*, 468–483, doi:10.1093/gbe/evt019.
2. Richards, E.J.; Ausubel, F.M. Isolation of a higher eukaryotic telomere from Arabidopsis thaliana. *Cell* **1988**, *53*, 127–136, doi:10.1016/0092-8674(88)90494-1.
3. Sýkorová, E.; Lim, K.Y.; Kunická, Z.; Chase, M.W.; Bennett, M.D.; Fajkus, J.; Leitch, A.R. Telomere variability in the monocotyledonous plant order Asparagales. *Proc. R. Soc. Lond. B* **2003**, *270*, 1893–1904, doi:10.1098/rspb.2003.2446.
4. Petracek, M.E.; Lefebvre, P.A.; Silflow, C.D.; Berman, J. Chlamydomonas telomere sequences are A+T-rich but contain three consecutive G-C base pairs. *Proceedings of the National Academy of Sciences* **1990**, *87*, 8222–8226, doi:10.1073/pnas.87.21.8222.

5. Tran, T.D.; Cao, H.X.; Jovtchev, G.; Neumann, P.; Novák, P.; Fojtová, M.; Vu, G.T.H.; Macas, J.; Fajkus, J.; Schubert, I.; et al. Centromere and telomere sequence alterations reflect the rapid genome evolution within the carnivorous plant genus *Genlisea*. *Plant J* **2015**, *84*, 1087–1099, doi:10.1111/tpj.13058.
6. Fajkus, P.; Peška, V.; Sitová, Z.; Fulnečková, J.; Dvořáčková, M.; Gogela, R.; Sýkorová, E.; Hapala, J.; Fajkus, J. *Allium* telomeres unmasked: the unusual telomeric sequence (CTCGGTTATGGG) $_n$ is synthesized by telomerase. *Plant J* **2016**, *85*, 337–347, doi:10.1111/tpj.13115.
7. Fulnečková, J.; Hasíková, T.; Fajkus, J.; Lukešová, A.; Eliáš, M.; Sýkorová, E. Dynamic Evolution of Telomeric Sequences in the Green Algal Order Chlamydomonadales. *Genome Biology and Evolution* **2012**, *4*, 248–264, doi:10.1093/gbe/evs007.
8. Matsuzaki, M.; Misumi, O.; Shin-i, T.; Maruyama, S.; Takahara, M.; Miyagishima, S.; Mori, T.; Nishida, K.; Yagisawa, F.; Nishida, K.; et al. Genome sequence of the ultrasmall unicellular red alga Cyanidioschyzon merolae 10D. *Nature* **2004**, *428*, 653–657, doi:10.1038/nature02398.
9. Peška, V.; Fajkus, P.; Fojtová, M.; Dvořáčková, M.; Hapala, J.; Dvořáček, V.; Polanská, P.; Leitch, A.R.; Sýkorová, E.; Fajkus, J. Characterisation of an unusual telomere motif (TTTTTTAGGG) $_n$ in the plant *Cestrum elegans* (Solanaceae), a species with a large genome. *Plant J* **2015**, *82*, 644–654, doi:10.1111/tpj.12839.
10. Moyzis, Robert K The Human Telomere. **1991**, *265*, 48–57.
11. Sahara, K.; Marec, F.; Traut, W. TTAGG Telomeric Repeats in Chromosomes of Some Insects and Other Arthropods. *Chromosome Research* **1999**, *7*, 449–460, doi:10.1023/A:1009297729547.
12. Müller, F.; Wicky, C.; Spicher, A.; Tobler, H. New telomere formation after developmentally regulated chromosomal breakage during the process of chromatin diminution in ascaris lumbricoides. *Cell* **1991**, *67*, 815–822, doi:10.1016/0092-8674(91)90076-B.
13. Mravinac, B.; Meštrović, N.; Čavrak, V.V.; Plohl, M. TCAGG, an alternative telomeric sequence in insects. *Chromosoma* **2011**, *120*, 367–376, doi:10.1007/s00412-011-0317-x.
